# Long‐term care facilities' response to the COVID‐19 pandemic: A protocol of a cross‐sectional, multi‐site, international survey

**DOI:** 10.1002/nop2.1264

**Published:** 2022-06-05

**Authors:** Sameh Eltaybani, Haruno Suzuki, Ayumi Igarashi, Mariko Sakka, Yuko Amamiya, Noriko Yamamoto‐Mitani

**Affiliations:** ^1^ Department of Gerontological Home Care and Long‐term Care Nursing The University of Tokyo Tokyo Japan; ^2^ Department of Nursing, Faculty of Healthcare Sciences Chiba Prefectural University of Health Sciences Chiba Japan

**Keywords:** aged, coronavirus, COVID‐19, cross‐sectional studies, long‐term care

## Abstract

**Aim:**

To examine the response of long‐term care (LTC) residential facilities to the COVID‐19 pandemic worldwide, and the antecedents and outcomes of this response.

**Design:**

A protocol of a cross‐sectional survey.

**Methods:**

Two online questionnaires will be used to collect data from LTC residential facilities' managers and staff worldwide. Collected data include participants' socio‐demographic characteristics, facility‐related characteristics, facility response to the COVID‐19 pandemic, and possible influences of the pandemic on staff, residents, and residents' families. Data collection has started in April 2021. Data analyses will be conducted on the pooled sample and stratified by the type of facility, participants, or country if required. Multi‐level regression analysis will be considered to account for participants' data clustering in countries and facilities.

**Results:**

The data collection is ongoing. The findings would guide policy‐makers and healthcare organizations to reform their protocols for the best interest of facilities, staff, residents, and residents' families.

## 1 INTRODUCTION

The recent pandemic of respiratory illness caused by the novel coronavirus (SARS‐CoV‐2) possesses a recognized risk on long‐term care (LTC) residential facilities' residents and staff (Thompson et al., 2020). Residents' old age, frailty, and comorbidities, and sharing the same sources of air, food, water, caregivers, and medical care, make them more vulnerable to be affected by the novel coronavirus disease (COVID‐19) (Bianchetti et al., 2020; Docherty et al., 2020; Li et al., 2020; Shi et al., 2020; Smilkov et al., 2014; Strausbaugh et al., 2003; Wang et al., 2020; Zheng et al., 2020). Further, direct, close, and frequent contact between staff and residents is inevitable, which makes the spread of respiratory infectious agents easier among staff and residents, particularly when there is a shortage of personal protective equipment, which was a frequently reported problem, particularly at the beginning of the pandemic (Blanco‐Donoso et al., 2021; Nyashanu et al., 2020; Ouslander & Grabowski, 2020). Published research showed that the COVID‐19 pandemic negatively affected LTC residential facilities' staff (Blanco‐Donoso et al., 2021; White et al., 2021), residents (El Haj et al., 2020; Levere et al., 2021), and residents' families (O’Caoimh et al., 2020; Wammes et al., 2020).

Several guidelines have been published to tackle the pandemic proliferation in LTC facilities. These guidelines centre around establishing monitoring systems; mandating the use of personal protective equipment; keeping physical distance; cleaning, disinfection, and respiratory hygiene; and providing sick leave compensation for staff (Rios et al., 2020). Measures implemented to combat the pandemic were also reported in many countries (International Long‐Term Care Policy Network, n.d.). Nevertheless, there is a lack of research‐driven data on how LTC facilities responded to the COVID‐19 pandemic in several countries, as well as factors associated with and outcomes of this response. There is also a lack of data on the association between the country profile (e.g., demography, economic status) and LTC facilities' response to the pandemic and outcomes of this response. Therefore, we sought to conduct an international study to examine how LTC facilities responded to the COVID‐19 pandemic, and factors associated with and outcome related to this response. The current manuscript describes the protocol of this proposed study.

### 1.1 Aims, research questions, and research hypotheses

The aim of this international study is three‐fold.

- To examine how LTC residential facilities responded to the COVID‐19 pandemic. In the current study, LTC facilities' response refers to the actions taken by LTC facilities to combat the infectious pandemic and mitigate its effect on residents, residents' families, and staff.
- To identify predictors of LTC residential facilities' response to the COVID‐19 pandemic. In the current study, predictors of LTC facilities' response are variables that would explain the ampleness or sufficiency of LTC facilities' response to the pandemic (i.e., variables that may explain the variability in LTC facilities' response to the infectious pandemic). In the present study, these variables are of three types: country‐, facility‐, and staff‐related variables.
- To examine how LTC residential facilities' response to the COVID‐19 pandemic influenced facilities' staff, residents, and residents' families. In the current study, the influence of the pandemic refers to the reported difference between “during the pandemic” and “before the pandemic” statuses of certain variables. In the present study, these variables are of three types: staff‐, resident‐, and residents' family‐related variables.

The specific research questions of the current study are as follows.

- How did LTC residential facilities respond to the COVID‐19 pandemic?
- What are the predictors of LTC residential facilities' response to the COVID‐19 pandemic?
- How did LTC facilities' response to the COVID‐19 pandemic influence their staff, residents, and residents' families?

**FIGURE 1:**
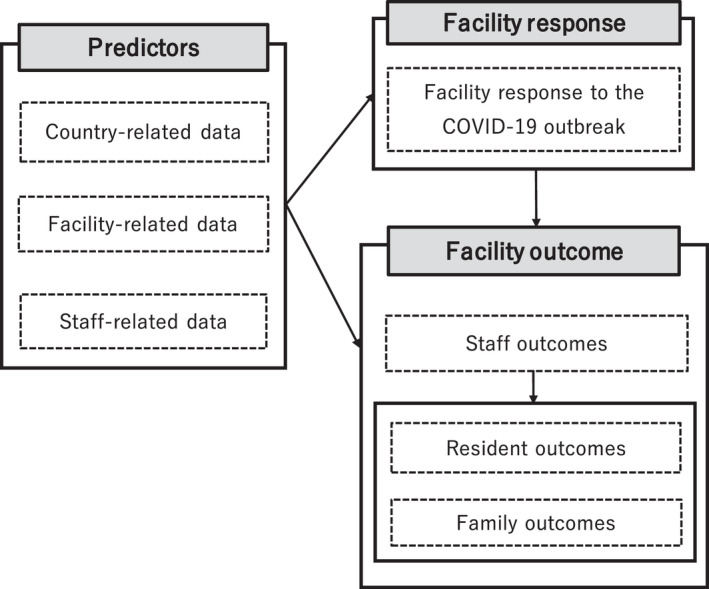
Conceptual framework of the study

Three hypotheses were set for the current study (Figure 1):

- LTC residential facilities' response to the COVID‐19 pandemic varies across and in countries.
- LTC residential facilities' response to the COVID‐19 pandemic is related to/may be explained by country‐, facility‐, and staff‐related factors.
- LTC residential facilities' response to the COVID‐19 pandemic influences staff, residents', and residents' families' outcomes.

## 2 MATERIALS AND METHODS

### 2.1 Research design

This is a protocol of a cross‐sectional, multi‐site, international, online survey.

### 2.2 Study settings

The study will be conducted in a convenience sample of LTC residential facilities anywhere in the world that could adhere to the study protocol. A country will be eligible for participation if it includes at least 10 LTC residential facilities. To date, 15 countries have participated in the study: Australia, Brazil, Egypt, Hong Kong, Indonesia, Japan, Norway, Portugal, Saudi Arabia, South Korea, Spain, Thailand, Turkey, the United Kingdom, and the United States of America. Research collaborators in each country will be responsible for recruiting LTC facilities for participation in the study (see Research Collaborators section below). The literature shows dramatic variations in the concept of LTC across and in countries and sectors in terms of goals, givers, activities, and target groups (Dyer et al., 2019; Sanford et al., 2015; Siegel et al., 2019; Tolson et al., 2013). Variations in country‐specific terminologies, formalization, and structure of LTC services, and potentially vague distinctions between types of LTC provision add to the complexities of establishing standardized language for residential LTC in a given country (Siegel et al., 2019). Equally, there are variations in the qualifications of carers. For instance, the term “nurse” in some countries is a title protected by statute, whereas in other countries, the term can include paid unlicenced assistive personnel or carers (Siegel et al., 2019). Such variations threaten the sample homogeneity in international research on LTC. Given that comparing types of LTC facilities is beyond the aim of the current study, we define LTC facilities as designed institutions that give formal (from paid staff) accommodation and health or social LTC services for older people. Health services include but are not limited to measuring vital signs, medical assessment, and assistance with medication. Social services include but are not limited to promoting social interaction, assessing psycho‐social–emotional needs, and facilitating contact between residents and community resources. In the current study, LTC residential facilities include but are not limited to skilled nursing facilities, nursing homes, assisted living facilities, and geriatric homes. For the purpose of the current study, the term LTC residential facilities does not include home‐based long‐term care, community centres, adult daycare facilities, respite care, or short‐stay rehabilitation. Facilities that meet the above definition will be eligible for inclusion if they fulfil the following criteria:

- Operating since October 2019 or earlier. This is to ensure that the facility has been operating for at least 3 months before the announcement of the COVID‐19 pandemic as a Public Health Emergency of International Concern in January 2020 (World Health Organization, 2020) in order to allow comparing “during the pandemic” and “before the pandemic” statuses.
- Having at least one nurse, whether as a manager or a direct care staff member (a nurse is a licenced healthcare professional who undertakes remunerated work for which formal nursing education is required). We assume that the scope of care and type of residents would vary among LTC facilities with and without a nurse. Despite the absence of strong evidence to support this assumption, the authors believe this inclusion criterion would increase the homogeneity of the study sample.
- A facility that agrees to participate in the study.

### 2.3 Study participants

This study will include participating facilities' managers and direct care staff. Participants will be recruited by research collaborators in each country (see Research Collaborators section). If the facility has more than one manager, the one who is in direct contact with the facility staff and residents will be asked to fill out the questionnaire; that is, one manager per facility. The staff questionnaire targets only healthcare professionals who give daily direct care to residents in the facility (e.g., nurses, care workers); the categories of included professionals will be judged by researchers in each country based on the country and facility regulations. The inclusion criteria of staff are a healthcare staff member who: (i) has been working at the facility during the calendar year 2020 whether on a part‐time or full‐time basis, (ii) is available at the facility at the time of data collection, and (iii) agrees to participate in the study.

### 2.4 Sampling and sample size

A convenience sampling approach will be used in this study. The rationale for adopting the convenience sampling technique is three‐fold. First, using random sampling necessitates having a sampling framework (e.g., a database of all LTC facilities in each country); such a framework might be missing or difficult to obtain in many countries, particularly in developing countries. Existing databases may not give complete information that allows researchers to judge the facility’s eligibility for the current study. Therefore, adopting a convenience sampling approach facilitates the conduct of the study by enabling researchers to involve facilities they already know as being eligible. Second, the current research is self‐supported at local study settings, and therefore, no commuting fees compensation will be given to research collaborators. Convenience sampling allows recruiting easy‐to‐access facilities and limits the need to commute to distant areas. Third, the workload in most LTC facilities worldwide has been increasing during the pandemic. This lowers facilities’ tendency to agree to participate in the research, particularly when the facilities are not acquainted with the researchers. Convenience sampling allows researchers to involve facilities with which they already have connections, which saves the time and efforts of research collaborators.

In terms of sample size, there will be no restrictions on the number of countries, facilities, or participants. To minimize the demerits of the convenience‐based sampling approach and increase the representativeness of the sample, we will attempt to include countries from the six continents and increase the homogeneity of the sample by pre‐defining eligibility criteria for both settings and participants. Besides, we will strive to include at least 10 facilities and at least 100 staff members from each country. Specifying a minimum required sample per country was set to prevent under‐ or over‐representation of any country in the overall sample. The minimum required sample per country was decided after a thorough discussion between the research management team and research collaborators in participating countries. The sample size of each country will be reported in the final report and will be considered before pooling data from different countries.

### 2.5 Measurements

Save for the country‐related data, data of the current study are self‐reported data collected using two questionnaires: the managers' questionnaire and the staff questionnaire. Questionnaires will collect data about the situation before and during the COVID‐19 pandemic. The COVID‐19 pandemic was announced to be a Public Health Emergency of International Concern on January 30, 2020 (World Health Organization, 2020). Therefore, the current study uses the term “*before the pandemic*” to refer to the period before January 2020 and the term “*during the pandemic*” to refer to the period from January 2020 to the date of filling the questionnaire.

#### 2.5.1 Facilities' response to the COVID‐19 pandemic

Based on a literature review, the adequacy of LTC facilities' response to the COVID‐19 pandemic will be assessed in terms of 12 dimensions: testing and screening, personal protective equipment, policy for staff shortage, education and training, psychological and mental support, resident‐family connectedness, visitation restriction, social distancing, separation of suspected and confirmed cases, recreational activities, dealing with new admissions, and promptness of the facility’s response. Questions related to each dimension will be given a score of 0, 1, or 2, where 0 indicates no response, 1 indicates minimal or insufficient response, and 2 indicates ample or intense response. The sum score of all items represents the overall adequacy of the facility’s response. Table 1 shows items used to assess LTC facilities’ response to the COVID‐19 pandemic and the response options and scoring of each item.

**TABLE 1:** Questions used to assess long‐term care facilities' response to the COVID‐19 pandemic

Dimension	Concept	Question	Scoring	Response options
1. Testing and screening	1. Active screening for residents^a^	During the COVID‐19 pandemic, residents were regularly assessed for fever and symptoms of respiratory infection (e.g., cough, shortness of breath)	2	Yes, regularly assessed
			1	Yes, assessed but not regularly
			0	No
	2. Active screening for staff^a^	During the COVID‐19 pandemic, staff were regularly assessed for fever and symptoms of respiratory infection (e.g., cough, shortness of breath)	2	Yes, regularly assessed
			1	Yes, assessed but not regularly
			0	No
	3. Having testing criteria^b^	During the COVID‐19 pandemic, the facility had criteria based on which novel Coronavirus testing for the staff and residents can be requested	2	Yes, we had such criteria early in the pandemic (in 2 months of the pandemic)
			1	Yes, but we had such criteria late in the pandemic
			0	No, we did not have such criteria
2. Personal protective equipment	4. Sufficiency of personal protective equipment^a^	During the COVID‐19 pandemic, there was shortage of personal protective equipment (e.g., gowns and face masks) and disinfectants in the facility	2	No, we did not face any problem
			1	Yes, but it was a temporarily, minor issue
			0	Yes, and it was a big problem for a long time
3. Policy for staff shortage	5. Policy to mitigate staff shortage^b^	During the COVID‐19 pandemic, the facility had a policy that describes how to mitigate possible staffing shortage	2	Yes, we had this policy early in the pandemic (in 2 months of the pandemic)
			1	Yes, but we had this policy late in the pandemic
			0	No, we did not have such a policy
4. Education and training	6. Staff education^a^	During the COVID‐19 pandemic, I have participated in a training course, workshop, or training program related to COVID‐19	2	Yes, and this education was comprehensive
			1	Yes, but this education was minimal
			0	No, I have not received any education related to COVID‐19
	7. Resident education^b^	During the COVID‐19 pandemic, the facility given education about COVID‐19 to residents	2	Yes, and this education was frequent and ample
			1	Yes, but this education was minimal or infrequent
			0	No education was given to residents about COVID‐19
	8. Family education^b^	During the COVID‐19 pandemic, the facility given education about COVID‐19 to residents' families	2	Yes, and this education was frequent and ample
			1	Yes, but this education was minimal or infrequent
			0	No education was given to families about COVID‐19
5. Psychological and mental support	9. Psychological and mental support to staff^a^	During the COVID‐19 pandemic, how do you evaluate the psychological support you received from the facility during COVID‐19 pandemic?	2	Lots of support
			1	Minimal support
			0	No support at all
	10. Psychological and mental support to residents^a^	During the COVID‐19 pandemic, how do you evaluate the psychological support given to the residents during COVID‐19 pandemic?	2	Lots of support
			1	Minimal support
			0	No support at all
6. Resident‐family connectedness	11. Keeping resident‐family connectedness^b^	During the COVID‐19 pandemic, the facility implemented some strategies to keep the resident‐family connectedness (e.g., by phone, Skype, Zoom, or any other method)	2	Yes, there was a frequent use of such measures
			1	Yes, but the use of such measures was minimal
			0	No, we did not implement such strategies
7. Visitation restriction	12. Restrictions on visits to facility^b^	During the COVID‐19 pandemic, the facility restricted visitors from entering the facility	2	Yes, visitation was completely restricted
			1	Visitation was allowed but less frequently and for shorter time
			0	No restrictions were applied on visitation
8. Social distancing	13. Application of social distance^a^	During the COVID‐19 pandemic, how often was the facility applying social/physical distancing (at least 1 meter/≥3 feet/about 1 arm length)?^c^	4	Always
			3	Often
			2	Sometimes
			1	Seldom
			0	Never
9. Separation of suspected and confirmed cases	14. Availability of private isolation rooms^b^	The facility had private rooms for resident quarantine/isolation when required	2	Yes, and they were enough for residents who needed isolation
			1	Yes, but they were not enough for residents who needed isolation
			0	No, the facility did not have any private room for resident isolation
	15. Sick leaves for staff^a^	The facility had a non‐punitive, flexible policy for staff sick leave (for example, when a staff member is sick or suspected to have COVID‐19)	2	Yes, we had such a policy early in the pandemic (in 2 months of the pandemic)
			1	Yes, but we had such a policy late in the pandemic
			0	No, we did not have such a policy
			0	I do not know^d^
10. Recreational activities	16. Restrictions on recreational activities^a^	During the COVID‐19 pandemic, the facility applied measures to prevent the spread of the novel Coronavirus infection when conducting recreational activities, such as restricting holding recreational activities, decreasing their frequency, or shortening their times.	2	Yes, such measures were applied early in the pandemic (in 2 months of the pandemic)
			1	Yes, but such measures were applied late in the pandemic
			0	No, such measures were not applied at all
11. Dealing with new admissions	17. New admission‐related policy^a^	During the COVID‐19 pandemic, the facility had a policy that describes how to deal with residents who are newly admitted to the facility to prevent the spread of the novel coronavirus infection	2	Yes, we had this policy early in the pandemic (in 2 months of the pandemic)
			1	Yes, but we had this policy late in the pandemic
			0	No, we did not have such a policy
			0	I do not know^d^
12. Promptness of the facility's response	18. Quickness in applying infection countermeasures^a^	In your opinion, how fast was your facility in implementing serious countermeasures to prevent and manage COVID‐19 pandemic (for example, applying restrictions on visits and recreational activities, providing education and training on COVID‐19, and using of personal protective equipment more than before)?^c^	4	Immediate (in 1 month of the pandemic)
			3	Quick (in 2 months of the pandemic)
			2	Slow (in 3–4 months of the pandemic)
			1	Late (after 4 months of the pandemic)
			0	To date, no serious countermeasures were taken

^a^ Data to be collected from facility staff. When computing a facility‐level score, responses will be aggregated at the facility‐level using the group mean.

^b^ Data to be collected from facility managers.

^c^ When computing a facility‐level score, score of this question will be divided by 2 to obtain a 0–2 score so that all questions would have the same weight.

^d^ When computing a facility‐level score, “I do not know” will be scored 0 based on the premises that even if policies exist, “I do not know” implies that policies were not put in action.

#### 2.5.2 Country‐related data

Data related to participating countries' demographic characteristics (e.g., proportion of older people), economic status (e.g., gross domestic product [GDP] per capita), healthcare status (e.g., health professionals‐to‐population ratio), and COVID‐19‐related statistics (e.g., COVID‐19 containment and health index) will be gathered from publicly available data. Data sources include but are not limited to the World Health Organization and the World Bank.

#### 2.5.3 Facility‐related data

The survey will collect data about manager’s characteristics (e.g., qualification), staffing pattern in the facility (e.g., staff‐to‐resident ratio), facility characteristics (e.g., facility size), and residents’ characteristics (e.g., comorbidities). The survey will also collect data about the situation before the COVID‐19 pandemic, such as the presence of infection control education and the presence of airborne infection‐related policy.

#### 2.5.4 Staff‐related data

Participating staff’s demography, qualification, work experience, motivation to work with older people, and source and ease of getting information about COVID‐19 will be elicited. The survey will also assess whether the participant is working across different facilities, has experienced social discrimination because of the job, lives with an older person, and whether any household members tested positive for SARS‐CoV‐2.

#### 2.5.5 Staff outcomes

The survey will examine the impact of the pandemic on staff absenteeism, shortage, turnover, intention to leave the job, workload, work stress, work satisfaction, work‐life balance, and the frequency of committing medication errors. Each of these outcomes will be assessed using a single item question, and responses are elicited on a 5‐level Likert scale. An example item is, “Compared to the situation before the COVID‐19 pandemic, your work‐life balance increased,” with five response options: “5 = *Strongly agree*,” “4 = *Somewhat agree*,” “3 = *Undecided*,” “2 = *Somewhat disagree*,” and “1 = *Strongly disagree*.” Further, the number of staff who tested positive for SARS‐CoV‐2 and those quarantined or passed away because of COVID‐19 will be assessed.

#### 2.5.6 Residents' outcomes

The survey will examine the impact of the pandemic on residents' fall rate, aggressive behaviours, feeling of loneliness and social isolation, feeling down and hopeless, physical condition, skin problems, and cognitive status. Each of these outcomes will be assessed using a single item (e.g., Compared to the situation before the COVID‐19 pandemic, residents' aggressive behaviours increased), and responses will be elicited on a 5‐level Likert scale: “5 = *Strongly agree*,” “4 = *Somewhat agree*,” “3 = *Undecided*,” “2 = *Somewhat disagree*,” and “1 = *Strongly disagree*.” The number of residents who tested positive for SARS‐CoV‐2 and those hospitalized or passed away because of COVID‐19 will also be assessed. In the current study, residents' outcomes were assessed by managers and staff instead of residents themselves for two reasons. First, since researchers were not allowed to enter LTC facilities in many countries due to the pandemic, and data would be collected only online, involving residents in the current study would require the facility manager or staff to recruit residents, explain to them the purpose of the study, and help them in responding to questions when needed. This would place additional efforts and load on staff and might negatively affect facilities and staff willing to participate in the study. Second, several research collaborators of the current study reported that involving residents would substantially lengthen the time needed for getting Research Ethics Committee approval in their countries, which threatens the feasibility of the data collection.

#### 2.5.7 Residents' families' outcomes

The survey will examine the impact of the pandemic on the frequency of making complaints by residents' families and their aggressive behaviours. Each of these outcomes will be assessed using a single item (e.g., *Compared to the situation before the COVID‐19 pandemic, the frequency of making complaints by residents' families increased*); response options are “5 = *Strongly agree*,” “4 = *Somewhat agree*,” “3 = *Undecided*,” “2 = *Somewhat disagree*,” and “1 = *Strongly disagree*.” Like residents' outcomes, family outcomes were assessed by managers and staff instead of residents' families themselves for two reasons. First, since families were refrained from visiting their relatives in LTC facilities in many countries amid the pandemic, it is highly probable that researchers will not have the opportunity to meet residents' families and will not be able to recruit them for participation. Requesting facility staff to carry out the recruitment process would place additional efforts and load on already loaded and busy staff. Second, several research collaborators of the current study reported that involving residents' families would substantially lengthen the time needed for getting Research Ethics Committee approval in their countries, which threatens the feasibility of the data collection.

### 2.6 Validation of the questionnaires

The questionnaires were developed in the English language. An international panel of researchers and practitioners was invited via e‐mail to validate and give suggestions to improve the content of the questionnaires, the wording and formatting of the questions, and the adequacy of the response options. Due to the lack of time to conduct a Delphi process, the validation process took place through online discussions with experts. The survey was made to fit the health care and LTC contexts of all participating countries. Questionnaires were then translated into each participating country’s local language by a professional translation company, which adopts three stages of translation: translation by a target language native translator, proofreading by an original language native translator, and a quality check by a translation manager. Further, at least one research collaborator in each participating country confirmed the semantic, idiomatic, experiential, and conceptual equivalence between the English and translated versions of the questionnaires and made modifications if needed.

### 2.7 Research collaborators

**FIGURE 2:**
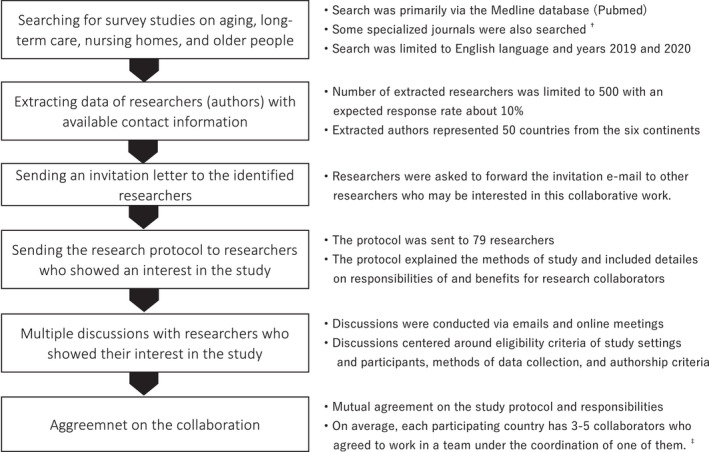
Recruiting research collaborators. † Examples of searched specialized journals are *Age and Aging*, *Geriatrics & Gerontology International*, *Journal of the American Geriatrics Society*, and *International Journal of Older People Nursing*. ‡ In each participating country, research collaborators' work is coordinated by a team coordinator (one of the country's collaborators) to prevent overlapping at the data collection sites, to minimize collaborators' workload, and to facilitate communication between the research managing team and research collaborators (the team coordinator acts as a contact point for each country's team)

The research managing team of the current study is composed of the authors of the current manuscript, with the first author being the principal investigator. Due to the nature of the present study, the research managing team recruited research collaborators worldwide. Figure 2 illustrates how research collaborators were recruited. Table 2 summarizes the roles of the research managing team and research collaborators.

**TABLE 2:** Responsibilities of the research managing team and research collaborators

Responsibilities of the research managing team
Writing the research protocol Constructing the data collection instruments Translating the data collection instruments into each participating country’s local language Recruiting research collaborators Sending the research protocol and data collection instruments to research collaborators Data management, curation, and analysis Announcement of results (e.g., manuscript publication, conference presentation)
Responsibilities of research collaborators
Recruiting long‐term care facilities in their countries Obtaining necessary approval to conduct the study from each facility Providing a detailed description of the study purpose and methods to potential participants and answering their questions when needed Ensuring the eligibility of study settings and study participants according to the study protocol Distributing the questionnaires to study participants Providing the research managing team with required information about study settings If paper questionnaires are used, Collecting the questionnaires from participants Entering the participants' responses into the online form Keeping the collected questionnaires for at least 5 years (or longer according to each participating country’s local regulations) after research publication

### 2.8 Data collection

Data will be collected using an online survey. The online survey was designed in a way that accessing, filling in, and submitting the questionnaire required basic computer literacy. The survey was created on SurveyMonkey®. The survey items were grouped into several sequential screens rather than being presented on a single, lengthy webpage. Turning forward and backward among screens is allowed through *Next* and *Previous* buttons. This design, which resembles completing a paper survey, aimed to prevent participants from scrolling through a lengthy page and potentially getting lost. Research collaborators may distribute the survey links to study participants in any convenient way, such as facility intranet or personal e‐mail. Paper questionnaires were also made available to be used if needed according to each site’s individual needs, such as limited online access or unfamiliarity with online surveys. The data collection has been ongoing since April 2021.

### 2.9 Data management and analysis

Collected data will be stored in an encrypted drive and will be accessed only by the research management team to ensure data confidentiality. Spreadsheets and SPSS will be used for data coding, cleaning, and analysis. Missing data will be analysed (e.g., using the Little’s missed completely at random test) and dealt with according to their pattern (Papageorgiou et al., 2018; Sainani, 2015). Data collected from the staff will be aggregated at the facility level to conduct facility‐level analysis. Descriptive and inferential statistical analyses will be conducted on the pooled sample and stratified by the type of facility, participants, or country if required. Multi‐level regression analysis will be considered to account for data clustering.

### 2.10 Ethical considerations

The Research Ethics Committee of the Graduate School of Medicine, The University of Tokyo, Japan, approved the current study (number: 2020329NI). Research collaborators will obtain Research Ethics Committee and administrative approval from study settings, following their national regulations. The cover page of the questionnaire will explain the study’s purpose and assure anonymity, confidentiality, and the right to refuse to participate in the study. The cover letter will also state that the completion and return of the questionnaire would be regarded as consent for participation. Further, a question asking participants if they want to participate in the study will be included; only participants who respond “Yes, I agree” will be included in the analysis. No personal or facility identification (e.g., names, e‐mails, mobile or telephone numbers) are collected. Each participating facility will be anonymously coded using a 1‐ to 4‐digit code assigned by research collaborators at the time of the data collection; this code will be linked to each survey response whenever the participant responds.

## 3 AUTHORSHIP AND SECONDARY PUBLICATIONS

Researchers participating in international studies are often referred to as authors, collaborators, contributors, research group, or collaborative group, and an explicit distinction between researchers' roles is often lacking. The current protocol clearly defines participating researchers' roles and contributions to prevent possible conflicts or unethical authorship. The International Committee of Medical Journal Editors (2019) defines the authorship criteria of publications in medical journals. Table 3 shows how the current study’s research collaborators will meet these criteria to co‐author publications from the current study data. Research collaborators who do not meet any of these criteria will be acknowledged in the *Acknowledgement* section of the final report. Research collaborators may use data of the current study for secondary analysis, publishing in scientific journals, or presenting in academic conferences; requests are addressed to and should be approved in advance by the principal investigator. Approval may be given after a written mutual agreement on what data will be used and co‐authors of the secondary publication. Some requests may be declined to prevent duplicate and salami‐slicing publication. Research collaborators are not allowed to use any of the data collected in this study for any purpose without written approval from the principal investigator.

**TABLE 3:** Meeting the authorship criteria

The International Committee of Medical Journal Editors' authorship criteria (2019)	Research collaborators' contribution to meet the authorship criteria
(1) Substantial contributions to the conception or design of the work; or the acquisition, analysis, or interpretation of data for the work	Revise and confirm the semantic, idiomatic, experiential, and conceptual equivalence between the English and translated versions of the questionnaires and make modifications if needed Recruit and collect data from long‐term care facilities in their countries after obtaining necessary Research Ethics Committee and administrative approval, following their national regulations
(2) Drafting the work or revising it critically for important intellectual content	Write a summary report about the situation of the COVID‐19 pandemic in long‐term care facilities in their countries; a unified Situation Summary form was developed by the research managing team and will be used by all researchers
(3) Final approval of the version to be published	Complete a conflict‐of‐interest form^a^ Getting each manuscript reviewed and approved by all research collaborators before publication may not be feasible due to the large number of collaborators. Therefore, submitting a conflict‐of‐interest form to the principal investigator implies that the research collaborator: Is willing to be a co‐author of any manuscript published from this study, Authorizes the principal investigator to approve and submit any report from this study in any form with no need to get the research collaborator's approval of the version to be published, and Agrees to be accountable for all aspects of the work in ensuring that questions related to the accuracy or integrity of any part of the work are appropriately investigated and resolved
(4) Agreement to be accountable for all aspects of the work in ensuring that questions related to the accuracy or integrity of any part of the work are appropriately investigated and resolved	

^a^ The conflict‐of‐interest form specified by the International Committee of Medical Journal Editors (2019) will be used.

## 4 DISCUSSION

The current study is expected to identify strength and vulnerability areas in LTC residential facilities' response to the COVID‐19 pandemic, variables associated with better responses, and response actions associated with better outcomes. The findings, therefore, would give real‐world research‐driven data that may help countries, institutions, and staff improves their performance during future infection outbreaks. To the best of our knowledge, this would be among the first international studies on the topic. The results are expected to show deficiency and strength areas in LTC facilities' response to the pandemic, which would help managers and policy‐makers identify areas that need attention and improvement. The results are also expected to show which response actions were associated with better outcomes, which helps facilities with low resources prioritize their actions. Besides, the current study is expected to identify factors associated with better response and better outcomes, which may help the development of proactive strategies to achieve better response and better outcomes.

Study protocol publication enhances research transparency, reduces publication bias, and informs the scientific community about what studies are being done, which helps avoid duplication and better coordinate research efforts. Making study protocols publicly available also has the benefit of disseminating the most contemporary ideas concerning study design and data analysis (Ohtake & Childs, 2014). The current protocol elaborates on the methods and conducts of several steps that are not often reported in international surveys, such as recruitment of research collaborators, roles of the research managing team and research collaborators, the translation and cross‐country validation of the questionnaires, data management and storage, and how research collaborators meet the authorship criteria. A number of publications have elaborated on the opportunities and challenges of conducting international research (Bosch & Titus, 2009; Dusdal & Powell, 2021; Green et al., 2004; Pinho & Reeves, 2021; See, 2018). Nevertheless, up to the best of our knowledge, there are no internationally agreed‐upon guidelines to conduct or report international healthcare‐related surveys. Detailed reporting of the methods and conduct of international surveys and challenges experienced might help the development of such guidelines.

A major challenge in the current study is the period of data collection, which was planned to take up to 1 month. This period, however, was extended for 6 months for three reasons. First, obtaining Research Ethics Committee approval in some countries was lengthy and took up to 8 months, which delayed the beginning of the data collection in these countries. Second, many countries were hit by the third wave of the COVID‐19 pandemic at the time of the data collection, which disturbed the schedule of research collaborators and hindered accomplishing the data collection on time. Third, most LTC facilities were overwhelmed with the care process and vaccinating the residents, engaged in other research, or both, making many facilities decline participation in this research. Future research needs to consider these challenges in designing and planning international surveys in similar contexts.

### 4.1 Limitations

Certain limitations of the current study merit mention. First, as mentioned above, several issues had led to extending the period of data collection, which might hinder cross‐country comparisons. To account for this issue, the period of data collection in each country will be reported in the final report and will be considered before pooling data from different countries. Second, the current study was planned and commenced before the initiation of any COVID‐19 vaccination and before the global widespread of different SARS‐CoV‐2 variants. Therefore, the current study did not take into account the contribution of vaccination nor different variants on the studied outcomes. Third, despite the strict eligibility criteria adopted to recruit LTC facilities and participants for the current study, participating facilities and subjects might still show a degree of variation. The authors believe that such variability is inevitable, and its influence on the validity and generalizability of the results would be minimal. The current survey was developed taking into account that items could be broad enough to fit all types of LTC facilities in all participating counties, to be easily responded to by study participants, and to give useful and sufficient information for assessing the actual situation in each participating facility. Fourth, resident‐ and family‐related outcomes will be sought from the facility managers and staff instead of residents and families themselves, which may raise questions about the validity of these outcomes. Finally, different sources of response bias exist. This includes convenience sampling and recall bias, and subjectivity and social desirability of self‐reported data.

## AUTHOR CONTRIBUTIONS

Sameh Eltaybani: Conceptualization, Writing—Original Draft, Writing—Review and Editing, Project administration. Haruno Suzuki, Ayumi Igarashi, Mariko Sakka and Yuko Amamiya: Conceptualization, Writing—Original Draft, Writing—Review and Editing. Noriko Yamamoto‐Mitani: Conceptualization, Writing—Original Draft, Writing—Review and Editing, Supervision.

## CONFLICT OF INTEREST

None declared.

## PATIENT CONSENT

Not applicable for this research.

## Data Availability

Data sharing not applicable to this article as no datasets were generated or analysed for the current protocol.
